# Use of Almitrine and Inhaled Nitric Oxide in ARDS Due to COVID-19

**DOI:** 10.3389/fmed.2021.655763

**Published:** 2021-07-01

**Authors:** Driss Laghlam, Ghilas Rahoual, Julien Malvy, Philippe Estagnasié, Alain Brusset, Pierre Squara

**Affiliations:** Department of Cardiology and Critical Care, Clinique Ambroise Paré, Neuilly-sur-Seine, France

**Keywords:** acute respiratory distress syndrome, almitrine, nitric oxide, mechanical ventilation, COVID-19

## Abstract

**Introduction:** Severe acute respiratory syndrome coronavirus 2 (SARS-CoV-2) is manifested by an acute respiratory distress syndrome (ARDS) with intense inflammation and endothelial dysfunction leading to particularly severe hypoxemia. We hypothesized that an impaired hypoxic pulmonary vasoconstriction aggravates hypoxemia. The objective of the study was to test the effect of two pulmonary vasoactive drugs on patient oxygenation.

**Methods:** Observational, single-center, open-label study in one intensive care unit (ICU) of the Paris area, realized in April 2020. Eligible patients had coronavirus disease 2019 (COVID-19) and moderate to severe ARDS [arterial partial pressure of oxygen/fraction of inspired oxygen (PaO_2_/FiO_2_) <200 mmHg] despite conventional protective ventilation. Exclusion criteria included pulmonary artery hypertension defined by a pulmonary artery systolic pressure (PAPs) >45 mmHg. The assessment of oxygenation was based on PaO_2_/FiO_2_ at (1) baseline, then after (2) 30 min of inhaled nitric oxide (iNO) 10 ppm alone, then (3) 30 min combination of iNO + almitrine infusion 8 μg/kg/min, then (4) 30 min of almitrine infusion alone.

**Results:** Among 20 patients requiring mechanical ventilation during the study period, 12 met the inclusion criteria. Baseline PaO_2_/FiO_2_ was 146 ± 48 mmHg. When iNO was combined with almitrine, PaO_2_/FiO_2_ rose to 255 ± 90 mmHg (+80 ± 49%, *p* = 0.005), also after almitrine alone: 238 ± 98 mmHg (+67 ± 75%, *p* = 0.02), but not after iNO alone: 185 ± 73 mmHg (+30 ± 5%, *p* = 0.49). No adverse events related to almitrine infusion or iNO was observed.

**Conclusion:** Combining iNO and infused almitrine improved the short-term oxygenation in patients with COVID-19-related ARDS. This combination may be of interest when first-line therapies fail to restore adequate oxygenation. These findings argue for an impaired pulmonary hypoxic vasoconstriction in these patients.

## Introduction

The severe acute respiratory syndrome coronavirus 2 (SARS-CoV-2) pandemic, identified as coronavirus disease 2019 (COVID-19), has affected millions of people worldwide since December 2019, with a mortality rate close to 1%. Severe SARS-CoV-2 is manifested by an acute respiratory distress syndrome (ARDS) defined according to the Berlin criteria (1), leading to particularly prolonged mechanical ventilation. However, it has been advocated that the COVID-19 pneumonia is a specific disease with peculiar phenotypes: mainly that there is a dissociation between the severity of the hypoxemia and the respiratory mechanics (2). In addition, the vasculature is also particularly affected, including an endothelial dysfunction contributing to tissue damage (3).

Regardless of the etiology, the mortality of ARDS patients is improved when reducing the ventilator-induced lung injury (4), including protective ventilation at 6 mL/kg of predicted body weight (pbw) of tidal volume (VT) tolerating “permissive hypercapnia” (5, 6), residual functional capacity restoration by individual optimization of positive end-expiratory pressure (PEEP) (7), plateau pressure (Pplat) limitation ≤ 30 cmH_2_0, use of myorelaxants (8). Moreover, in cases of persisting severe hypoxemia [typically when the ratio of arterial partial pressure of oxygen to the fraction of inspired oxygen (PaO_2_/FiO_2_) is >150 mmHg], prone position (PP) sessions for at least 16 consecutive hours have proven beneficial (9). In the most severe forms, when PaO_2_/FiO_2_ <80 mmHg despite these interventions, and/or when mechanical ventilation becomes harmful due to high Pplat, venovenous extracorporeal membrane oxygenation (ECMO) can be proposed (10).

The main mechanism of hypoxemia in ARDS is an inflammation-induced intrapulmonary shunt caused by alveolar flooding and alveolar collapse due to a loss of surfactant (11). Hypoxic pulmonary vasoconstriction (HPV) is a homeostatic mechanism that is intrinsic to the pulmonary vasculature in response to alveolar hypoxia, shunting the blood flow away from the hypoxic territories (12). Consequently, pulmonary pressure has been shown early to have a strong negative prognostic value in ADRS (13).

Nitric oxide (NO) is a selective pulmonary arterial vasodilator. When NO is inhaled (iNO), it improves ventilation–perfusion ratios by preferentially redistributing blood flow to the ventilated areas. In ARDS patients, iNO improves gas exchange and both pulmonary arterial hypertension and right ventricular failure, which both have negative prognoses in ARDS (13–15). Given a fairly favorable benefit–risk ratio, the physiological effects of iNO can therefore justify its use in severe ARDS when optimized mechanical ventilation does not correct hypoxemia (15, 16).

On the other hand, the inflammation may alter the intrinsic mechanism of HPV (12), leading to the consideration of testing selective pulmonary vasoconstrictors. Almitrine is a peripheral chemoreceptor stimulant that has been reported to improve the oxygenation in ARDS patients by increasing hypoxic pulmonary vasoconstriction (17). Therefore, its use has been proposed to improve gas exchange in ARDS, alone or in combination with iNO (18, 19). Although the effect of these drugs is often transient and their effect on mortality has not been established to date, the rationale for using a combination of perfused vasoconstrictors and inhaled vasodilators is to improve the ventilation/perfusion ratio (V/Q) through selective vasoconstriction of pulmonary vessels perfusing non-aerated areas and selective vasodilation of pulmonary vessels perfusing aerated areas.

Given the peculiar severity of hypoxemia in COVID-19 lung injury, we hypothesized that endothelium dysfunction may alter the HPV. Then, iNO and almitrine could be tested to improve the V/Q. The aim of our study was to assess the effect of these drugs, alone and in combination, on the oxygenation of patients with moderate to severe ARDS due to COVID-19.

## Methods

This was an observational, single-center, open-label study in one intensive care unit (ICU) of the Paris area, realized in April 2020. The study was approved by the local ethics committee as a component of standard care. Patients and/or families were given information about the study. Following French regulations, all patients (or their relatives in case of death) were informed at discharge that the data collected during their stay could be anonymously used for scientific purpose and that they can ask to have their data erased.

### Patients

Eligible patients had COVID-19 (confirmed by RT-PCR on a nasopharyngeal sample) and ARDS according to the definition of the Berlin criteria (1). All had CT scans. Patients were included if they had moderate to severe ARDS (PaO_2_/FiO_2_ <200 mmHg) despite conventional treatment: effective sedation and curarization, protective ventilation at 6 mL/kg with optimized PEEP level to maintain Pplat ≤ 30 cmH_2_O, and had already at least one session of ventilation with PP; however, no patient was in PP at the time of the protocol. One patient was on venovenous ECMO at the time of the study. Exclusion criteria were known allergy to iNO and/or almitrine, pulmonary artery systolic pressure (PAPs) >45 mmHg, measured by a transthoracic echocardiography-Doppler standard examination.

### Measurements

All the measurements concerning ventilatory and hemodynamic variables were carried out during the protocol by a single operator in charge of the patient. As part of the standard care of ARDS, a radial or femoral arterial catheter was placed in all patients, allowing monitoring of the systemic arterial pressure and sampling for blood gas analysis, including lactate. A transthoracic echocardiography was performed to evaluate the left and right ventricular function. The presence of right-to-left shunting was systematically evaluated before the initiation of the protocol. The PAPs was estimated from the flow of tricuspid regurgitation during echocardiography using 4 × Vmax + 10 (representative of the mean right atrium pressure). Data of the mechanical ventilation were collected: VT, respiratory frequency, PEEP, PPlat, dynamic compliance, and driving pressure. The static compliance was calculated and according to the formula: VT/(Pplat-PEEP).

### Protocol

All patients were sedated, curarised, under assist-control ventilation with pure oxygen (FiO_2_ = 100%) throughout the complete protocol. The depth of sedation and curarization was controlled and unchanged. The ventilation parameters, vasopressors/inotropic posology, and fluid perfusion were planned to remain constant throughout the protocol.

A blood gas sample [including arterial pH, PaO_2_, partial pressure of carbon dioxide (PaCO_2_), lactate level] and an echocardiography examination with PAPs measurement were performed for each patient at (1) baseline, (2) after 30 min of iNO administration alone, (3) after 30 min of a combination of iNO + almitrine administration, and (4) after 30 min of almitrine alone. The iNO (KINOX®) was delivered continuously from a specific dispositive (Air Liquide, Paris, France) at a concentration of 10 ppm into the inspiratory limb of the ventilator. Almitrine (Vectarion®, Servier, Suresnes, France) was delivered intravenously *via* a central venous catheter at a concentration of 8 μg/kg/min. We did not plan any washout since the sequence of the protocol avoided any unexpected mix. Also, since we were interested in studying the combination of drugs, we did not plan a return to baseline between the changes of the regimen.

### Statistical Analysis

Categorical variables are reported as numbers and proportions (%). Continuous data are reported as the mean ± standard deviation when normally distributed or median with interquartile ranges (25–75th) when not. Normal distribution was controlled by Shapiro tests. We used the χ2 test or Fisher exact test to compare categorical variables, the Mann–Whitney *U*-test to compare medians, and ANOVA to compare means. For all tests, *p* < 0.05 was considered statistically significant. All statistical analyses were performed with SPSS 25.0.

## Results

### Population Characteristics

Fifty-four patients required mechanical ventilation for pneumonia due to COVID-19 from the beginning of the pandemic and 20 during the study period; 12 of them met the inclusion criteria (five had PAPs >45 mmHg, and three had PaO_2_/FiO_2_ >200 mmHg). Among these 12 patients (Table 1), nine were men (75%), mean age was 71.8 ± 8.7 years old, and seven patients had diabetes mellitus (58%) and hypertension (58%). Only one patient was a smoker (8%) with a documented chronic obstructive pulmonary disease (COPD). Most patients (11/12, 92%) had left ventricular ejection fraction (LVEF) >50%; two patients had segmental pulmonary embolism without right ventricular failure (17%). The mean duration of the mechanical ventilation at the time of inclusion was 11.0 ± 8.3 days. All patients received norepinephrine during their hospitalization, but only five (42%) still received it during the protocol.

**Table 1:** Baseline characteristics.

	**Gender**	**Age**	**Day**	**CRP**	**D-dimer**	**Fibr**	**VT**	**RR**	**PEEP**	**DP**	**Compliance**	**VR**	**Lactate**	**NAD**
		**(years)**		**(mg/L)**	**(ng/mL)**	**(g/L)**	**(mL/kgpbw)**	**(per min)**	**(cmH_**2**_O)**	**(cmH_**2**_O)**	**(mL/CmH_**2**_O)**		**(mMol/L)**	**(mg/h)**
1	F	75	16	164	6,770	8.2	6	30	9	22	16	3.1	0.9	0
2	M	73	8	178	1,934	9.2	5.5	28	14	14	35	2.8	1.8	0.3
3	M	66	14	165	20,700	5.8	5	30	9	23	17	2.5	0.8	0
4	M	73	5	296	857	9.2	5.6	24	10	21	20	1.7	2	0
5	M	71	26	101	4,280	3.7	5.3	28	8	18	23	2.1	1.3	1
6^*^	F	76	15	298	671	6.5	3.6^*^	16	12	10	29	1.3	0.8	0
7	M	84	13	299	647	10	5.2	28	10	19	23	3.0	1.3	0
8	M	80	7	271	28,096	5.1	5.6	30	10	16	26	2.3	1.3	3
9	M	60	24	178	989	6.0	6	18	10	19	24	1.7	0.6	0
10	M	81	2	292	5,994	8.5	5.7	24	10	18	33	1.5	2.5	0.3
11	F	54	4	407	1,434	9.5	5.6	18	14	10	32	1.5	1.6	0
12	M	68	2	63	1,481	5.2	5.8	22	14	15	30	1.9	4	3.2
Mean		71.8	11.3	226	1,708	7.2	5.6	24.7	10	17.1	25.7	2.1	1.3	0
SD		8.7	8.1	99.5	(890–6,576)	2.1	(5.2–5.8)	5.1	(9.3–10.5)	4.3	6.2	0.6	(0.83–2)	(0–0.8)

*CRP, C-reactive protein; Day, days from intubation; DP, driving pressure; F, female; Fibr, fibrinogen; M, male; Mean, mean or median; NAD, norepinephrine; PEEP, positive end-expiratory pressure; RR, respiratory rate; SD, standard deviation or interquartile range; VR, ventilatory ratio; VT, tidal volume in kg per predicted body weight;*

* *ECMO VV blood flow rate 4.5 L/min, sweep gas flow rate 4 L/min; FiO_2_ = 60%*.

The percentage of lung involvement on CT scan was 50% (40–70%). There was no correlation between CT score and the response to any of the treatments: for iNO (*r* = 0.039; −0.55–0.60; *p* = 0.90), iNO + almitrine (*r* = −0.51; −0.84–0.092; *p* = 0.09), almitrine (*r* = −0.56; −0.86–0.02; *p* = 0.06).

The median transthoracic echocardiographic measurements at baseline were: tricuspid annular plane systolic excursion (TAPSE) = 19.5 (16–21) mm, velocity time integral left ventricular outflow tract (VTI LVOT) = 17 (15.8–18.5) cm, right/left ventricular ratio = 0.48 (0.42–0.6), PAPs = 38 (33–42.3) mmHg, LVEF = 55% (52–60%).

**Ventilatory pattern at baseline was**: VT = 5.6 (5.2–5.8) mL/kg, respiratory frequency = 24.6 ± 5.1/min, PEEP = 10 (9.3–10.5) cmH_2_O, Pplat = 27.9 ± 3.0 cmH_2_O, driving pressure = 17.1 ± 4.3 cmH_2_O, compliance = 25.6 ± 6.2 mL/cmH_2_O, PaO_2_/FiO_2_ =146 ± 48 mmHg, PCO_2_ = 52 ± 8.3 mmHg, ventilatory ratio = 2.1 ± 0.6.

### Protocol Results

**Figure 1:**
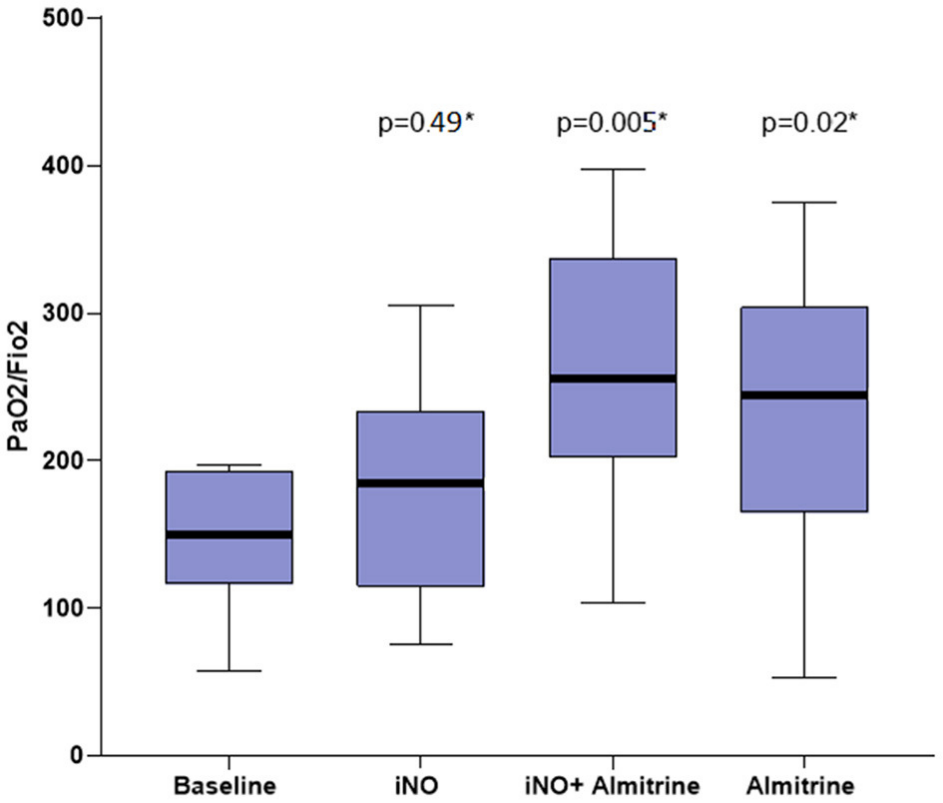
Arterial partial pressure of oxygen/fraction of inspired oxygen (PaO_2_/FiO_2_) evolution, *comparison from baseline.

Details of the evolution of ventilatory and hemodynamic variables are shown in Table 2. Evolution of PaO_2_/FiO_2_ is presented in Figure 1. After iNO, PaO_2_/FiO_2_ increased from 146 ± 48 mmHg to 185 ± 73 mmHg (+30 ± 35%, *p* = 0.49). After iNO combined with almitrine, PaO_2_/FiO_2_ increased significantly from baseline: 255 ± 90 mmHg, (+80 ± 49%, *p* = 0.005). With almitrine alone, PaO_2_/FiO_2_ was maintained significantly higher than that at baseline: 146 ± 48 to 238 ± 98 mmHg (+67 ± 75%, *p* = 0.02). The change in PaO_2_/FiO_2_ when iNO was stopped was not significant (238 ± 98 vs. 255 ± 90, *p* = 0.67). The PaO_2_/FiO_2_ increased by at least 20% in 50%, 92% and 75% of the patients after iNO, iNO + almitrine, and almitrine alone, respectively (Table 2). Six patients were poor responders (PaO_2_/FiO_2_ increase <20%) with iNO alone and four with almitrine alone, but only one was a poor responder to the combination of both drugs (patient 12). This patient was the only one responding better to iNO than to almitrine. Furthermore, when norepinephrine was withdrawn and ECMO was initiated, the protocol was restarted in this patient and he became a responder to the combination of both drugs.

**Table 2:** Ventilatory Pattern During the Protocol.

	***Baseline***					***iNO***						***iNO+almitrine***						***Almitrine***					
	***P/F***	***PaCo_**2**_***	***PAPs***	***ΔP***	***Cp***	***P/F***	***%***	***PaCo_**2**_***	***PAPs***	***ΔP***	***Cp***	***P/F***	***%***	***PaCo_**2**_***	***PAPs***	***ΔP***	***Cp***	***P/F***	***%***	***PaCo_**2**_***	***PAPs***	***ΔP***	***Cp***
1	131	66	43	22	16	103	0.8	57	39	23	16	233	1.8	54	46	21	17	302	2.3	62	46	20	18
2	178	54	38	14	35	291	1.6	55	33	14	35	341	1.9	51	38	14	35	375	2.1	52	38	14	35
3	57	59	38	23	17	97	1.7	53	35	21	19	104	1.8	53	46	23	17	53	0.9	59	48	23	17
4	189	43	37	21	20	170	0.9	46	35	20	21	261	1.4	46	46	19	22	214	1.1	53	48	18	23
5	137	50	45	18	23	237	1.7	47	43	21	20	327	2.4	48	42	19	22	289	2.1	48	47	18	23
6^*^	71	56	43	10	29	76	1.1	53	41	10	29	196	2.8	48	47	12	24	248	3.5	47	42	12	24
7	162	65	33	19	23	165	1.0	67	30	20	22	221	1.4	61	35	21	21	216	1.3	64	39	21	21
8	194	42	42	16	26	206	1.1	39	40	15	28	251	1.3	41	43	15	28	241	1.2	42	45	16	26
9	195	56	40	19	24	305	1.6	61	37	18	25	342	1.8	59	40	19	25	305	1.6	64	42	19	25
10	134	39	27	18	33	224	1.7	37	26	17	31	265	2.0	36	28	18	31	148	1.1	39	29	18	31
11	197	44	32	10	32	199	1.0	42	30	10	32	398	2.0	42	31	10	32	357	1.8	40	33	10	32
12	111	52	28	15	30	150	1.4	57	26	14	30	115	1.0	55	33	14	30	106	1.0	58	38	14	30
12^*^	93	50	30	15	21	104	1.1	51	31	14	23	194	2.1	48	31	14	23	96	1.03	51	30	13	25
M	146	52	37	17	25	185	1.30	51	35	17	25	255	1.80	50	39	17	25	238	1.67	52	41	17	25
SD	48	8.9	5.7	4.3	6.2	76	0.35	9.1	5.2	4.3	5.9	90	0.49	7.8	6.7	4.0	5.7	98	0.75	9.2	6.9	3.9	5.4

*P/F, arterial partial pressure of oxygen/fraction of inspired oxygen (PaO_2_/FiO_2_); ΔP, driving pressure; PaCO_2_, partial pressure of carbon dioxide; PAPs, pulmonary artery systolic pressure; Cp, compliance; M, mean or median; SD, standard deviation or interquartile range;*

* ECMO, 12*, same patient but after norepinephrine removal and ECMO VV implantation; %, percentage of PaO_2_/FiO_2_ changes after each step.

We found no relationship between the change in PaO_2_/FiO_2_ during the protocol and any of the ventilatory variables assessed at baseline. Pplat, driving pressure, compliance, arterial pH, and PCO_2_ levels did not change significantly during the trial. We observed no adverse events related to almitrine infusion or iNO. Hemodynamic variables remained comparable before and after the trial: arterial pressure (76.6 ± 12.6 vs. 74.8 ± 9.1 mmHg, *p* = 0.69), heart rate (91 ± 25 vs. 95/min ± 23 beats/min, *p* = 0.66), lactates [1.3 (0.9–1.9) vs. 1.6 (1.2–1.9) mMol/L, *p* = 0.60]. No patient developed acute cor pulmonale during the protocol.

The serum inflammation marker levels at admission, peak, and day of trial were respectively: C-reactive protein (CRP): 213 ± 105 mg/L, 333 ± 49 mg/L, 226 ± 99.5 mg/L; fibrinogen: 7.1 ± 2.1 g/L, 8.9 ± 1.3 g/L, 7.2 ± 2.1 g/L; D-dimer: 1,481 (989–5,994) ng/mL, 12,706 (3,522–22,948) ng/mL, 1,708 (890–6,576) ng/mL. We found no correlation between PaO_2_/FiO_2_ evolution after almitrine infusion and these marker serum levels. However, three patients had received interleukin (IL)-6 inhibitors that quickly decreased these markers' levels over time.

At the end of the protocol, the combination of iNO and almitrine was continued at the discretion of the physician in all patients. The median duration of mechanical ventilation was 36 (18–50) days. Five patients (42%) underwent a tracheostomy. The final mortality was 50% at 90 days (patients 3, 5, 7, 8, 10, and 12).

## Discussion

We found that the combination of iNO and intravenous almitrine infusion improved the short-term oxygenation of patients with ARDS due to COVID-19, even when they received moderate doses of norepinephrine. This immediate beneficial effect was obtained with moderate iNO concentrations (10 ppm) and almitrine (8 μg/kg/min). The use of higher doses of norepinephrine seems to alter the response to the combination of drugs. One patient (number 12) was not a responder when receiving more than 3 mg/h of norepinephrine and became a responder after norepinephrine withdrawal and ECMO initiation. No hemodynamic impairment occurred during our protocol study, with no significant modification in PAPs, heart rate, and plasma lactate level.

These results are consistent with past studies using invasive hemodynamic measurements in ARDS patients and confirmed the excellent tolerance of these drugs at these doses (19, 20). Some authors used higher doses of almitrine, up to 16 μg/kg/min, and they found that it could impair the loading condition of the right ventricle (21).

Almitrine improved the oxygenation of our patients alone or in combination. In contrast, the use of iNO alone did not allow a significant increase in PaO_2_/FiO_2_, and when the iNO was removed, the decrease in PaO_2_/FiO_2_ was again not significant. This argues for a moderate effect of iNO in our patients and, therefore, for an alteration of the expected HPV as a predominant mechanism of V/Q mismatch in COVID-19 ARDS. In order to further investigate this mechanism, larger studies with comparison of almitrine effects on COVID-19-related ARDS and other causes of ARDS could be interesting. Furthermore, we performed this protocol only on supine position. As almitrine and iNO are actually recommended in ARDS patients in cases of refractory hypoxemia despite PP (22), it should be interesting to investigate their effects in PP.

Recent meta-analysis on the application of iNO in ARDS has shown that iNO can improve short-term oxygenation, but it does not improve prognosis and has an increased risk of renal insufficiency (risk ratio between 1.55 and 1.59) (23–25). It needs to be kept in mind when the benefit–risk balance from the use of iNO arises.

These findings are in line with recently published studies. The same results were obtained at a lower dose of almitrine (2 μg/kg/min) associated or not with iNO in 19 COVID-19 patients with persistent refractory hypoxemia, with an increase of the median PaO_2_/FiO_2_ ratio from 79 (64–100) at baseline to 117 (81–167) after almitrine (*p* = 0.001) (26). Comparative results were found with infusion of 10 μg/kg/min of almitrine, associated in 75% of cases with iNO (10 ppm). Twenty-one patients (66%) were described as responders (increase of PaO_2_/FiO_2_ ratio ≥20% at the end of the infusion); the median PaO_2_/FiO_2_ ratio improvement was 39% (9–93%) and differed significantly between the responders and non-responders [67% (39–131%) vs. 6% (9–16%), respectively; *p* < 0.0001] (27). Some authors tested the dose effects of almitrine, and its infusion alone was associated with an improvement of PaO_2_/FiO_2_ ratio from 135 at baseline to 149 at 4 μg/kg/min and 215 at 12 μg/kg/min (*p* = 0.06) on 8/10 patients at the early phase of severe COVID-19 ARDS. In this study, three patients were on PP during the protocol and the amplitude of PaO_2_ increase was different according to the patient's position (PP vs. supine position) supposing that the combination of gravitational and pharmacogical effects was synergistic to improve the VA/Q mismatch (28). The effects of iNO (10 ppm) alone and in association with 10 μg/kg/min almitrine was also tested just after a prone session. Authors founds that the median of PaO_2_/FiO_2_ ratio increased from 102 (89–134) mmHg at baseline to 124 (108–146) mmHg after iNO (*p* = 0.13) and 180 (132–206) mmHg after iNO and almitrine (*p* < 0.01) but showed no correlation between the increase in oxygenation caused by iNO–almitrine combination and that caused by proning (29). Another uncontrolled study showed conflicting results. The use of iNO, almitrine, or both did not improve the oxygenation in 20 severe COVID-19 ARDS; however, the patients of this study also had more serious lung injury than those in our study (median PaO_2_/FiO_2_ = 106) (30).

HPV, also known as the Euler–Liljestrand mechanism, is a homeostatic mechanism in which the small pulmonary arteries constrict in the presence of low alveolar oxygen tension. In that situation, a mitochondrial sensor dynamically changes reactive oxygen species and redox couples in pulmonary artery smooth muscle cells, leading to activate voltage-gated calcium channels and to increase cytosolic calcium, causing vasoconstriction. It improves V/Q matching by redirecting the blood flow from poorly ventilated lung regions to normally ventilated lung regions (31). Many factors inhibit HPV, including increased cardiac output, hypocapnia, hypothermia, acidosis/alkalosis, and PEEP. Different diseases are also known to alter the physiological mechanism of HPV such as liver cirrhosis, COPD, and sepsis. Lastly, different drugs may also alter the HPV mechanisms including anesthetic agents, isoproterenol, calcium blockers, and vasodilators. Chloroquine was found to decrease HPV through a combination of vasodilator, anti-proliferative, and anti-autophagic effects (32). None of these factors were present at the time of the protocol, but it is impossible to eliminate an effect of one or several of them, especially chloroquine that was given to all our patients in the pre-intubation phase. Nevertheless, the most probable cause of impaired HPV is inflammation, which is severe in COVID-19 patients, as it was in all our patients (33). The direct mechanism of impaired HPV and inflammation/endothelial dysfunction is unknown but may be part of the endothelium dysfunction, a silent component of inflammation (34) particularly found in COVID-19 and predisposing patients to thrombosis and platelet activation (35). Indeed, endothelitis in lung vessels and others organs with the presence of viral elements within endothelial cells and an accumulation of inflammatory cells, with evidence of endothelial and inflammatory cell death, was found in COVID-19 patients (36).

Our study had several limitations. First, it was a monocentric study performed on a restricted number of patients. Then, the treatments were given in the same order, in the same dose for each patient, and not randomly. Furthermore, the design of the study did not allow us to assess whether this beneficial effect on oxygenation was sustained over time and/or may change the outcome. Despite these limitations, the homogeneous response to the protocol made it generalizable to all COVID-19 patients with moderate to severe V/Q mismatch, with few chances of being wrong.

## Conclusion

Combining 10 ppm of iNO and 8 μg/kg/min of infused almitrine improved the short-term oxygenation in patients with ARDS due to COVID-19. Impaired hypoxic pulmonary vasoconstriction due to major inflammation and endothelial dysfunction may be a preponderant mechanism of hypoxia of this pathology. This combination may be of interest when first-line therapies of ARDS fail to restore the oxygenation sufficiently.

## Data Availability Statement

The raw data supporting the conclusions of this article will be made available by the authors, without undue reservation.

## Ethics Statement

The studies involving human participants were reviewed and approved by CMC Ambroise Paré local ethics commitee. The patients/participants provided their written informed consent to participate in this study.

## Author Contributions

All authors listed have made a substantial, direct and intellectual contribution to the work, and approved it for publication.

## Conflict of Interest

The authors declare that the research was conducted in the absence of any commercial or financial relationships that could be construed as a potential conflict of interest.

## Abbreviations

ARDS: acute respiratory distress syndrome
ECMO: extracorporeal membrane oxygenation
FiO_2_: fraction of inspired oxygen
HPV: hypoxic pulmonary vasoconstriction
iNO: inhaled nitric oxide
PP: prone position
PaO_2_: arterial partial pressure of oxygen
PEEP: positive end-expiratory pressure
V/Q: ventilation/perfusion.
