# Supplementary figures and images for: Use of Almitrine and Inhaled Nitric Oxide in ARDS Due to COVID-19
Source: Front Med (Lausanne). 2021 Jul 1;8:655763. doi: 10.3389/fmed.2021.655763 (PMC8280335; doi:10.3389/fmed.2021.655763)

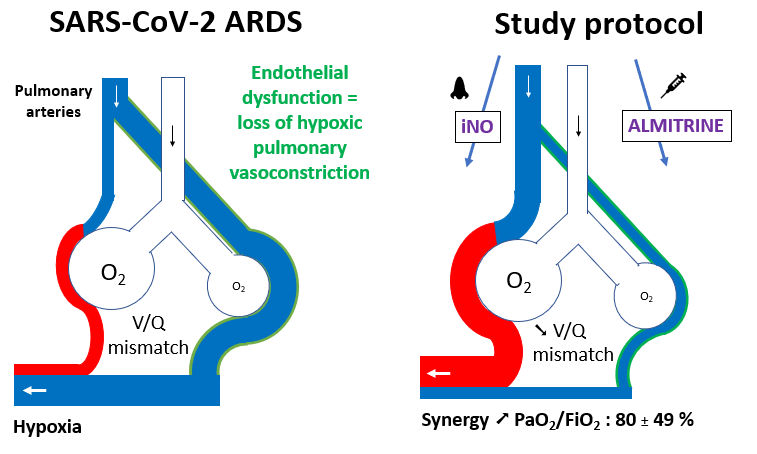

Supplement: Supplementary file 1 [file Image_1.TIF]
